# Application of Subspace Clustering in DNA Sequence Analysis

**DOI:** 10.1089/cmb.2015.0084

**Published:** 2015-10-01

**Authors:** Tim Wallace, Ali Sekmen, Xiaofei Wang

**Affiliations:** ^1^ Department of Computer Science, Tennessee State University, Nashville, Tennessee.; ^2^ Department of Biological Sciences, Tennessee State University, Nashville, Tennessee.

**Keywords:** algorithms, statistics

## Abstract

**Identification and clustering of orthologous genes plays an important role in developing evolutionary models such as validating convergent and divergent phylogeny and predicting functional proteins in newly sequenced species of unverified nucleotide protein mappings. Here, we introduce an application of subspace clustering as applied to orthologous gene sequences and discuss the initial results. The working hypothesis is based upon the concept that genetic changes between nucleotide sequences coding for proteins among selected species and groups may lie within a union of subspaces for clusters of the orthologous groups. Estimates for the subspace dimensions were computed for a small population sample. A series of experiments was performed to cluster randomly selected sequences. The experimental design allows for both false positives and false negatives, and estimates for the statistical significance are provided. The clustering results are consistent with the main hypothesis. A simple random mutation binary tree model is used to simulate speciation events that show the interdependence of the subspace rank versus time and mutation rates. The simple mutation model is found to be largely consistent with the observed subspace clustering singular value results. Our study indicates that the subspace clustering method may be applied in orthology analysis.**

## 1. Introduction

Identification of orthologous relationships among protein and nucleotide sequences is of wide interest for biological sequence analysis because such information provides insight into the molecular function and evolutionary history of these sequences. To date, many mathematical techniques have been utilized intensively and extensively in biological sequence analysis to perform diverse functions, ranging from sequence alignment to sequence clustering (e.g., survey Andreopoulos et al, 2009).

From a computational perspective, the two common methods of clustering of orthologous genes are three-way triangle relative reciprocal similarity and graph clustering used in NCBI clusters of orthologous groups (COG) and orthoMCL works (respectively in Wolf et al., 2012, and Li et al., 2003). Both of the aforementioned methods are well represented in the literature as used in orthologous protein classifications. The development of additional mathematical tools and theory for analysis of related sequences is an area of burgeoning interest. Examples may be found in the literature, such as in Kim and Lee (2006) and Viswanath and Madabhushi (2012).

We propose a new approach based upon a hypothesis that under evolutionary constraint, descendant sequences from a common ancestor share a mathematical subspace, as opposed to ambient space. In particular, we hypothesize that member nucleotide or peptide sequences lie within low dimensions within the ambient space of all sequences. Therefore, the subspace dimensions are a reflection of mutation and time elapsed since the speciation or duplication within the cluster.

In order to investigate the hypothesis, several sets of experiments and estimates of the *p*-values for the the null-hypothesis are computed. The nucleotide sequences data were taken from the reverse mapping provided by NUCOCOG, that is, the nucleotide sequences–based COG database of Meereis and Kaufmann (2008), relating the works of Wolf and Makarova (2012) at the NCBI. A simple interface was written to extract the raw nucleotide sequences for a given cluster identification number. The subspace clustering was initially performed on the nucleotide sequences. However, the subspace clustering was found to produce similar results with the forward mappings to amino acids forming proteins. The accuracy was found to be slightly less than the nucleotide clustering, presumably due to the degeneracy of the forward mapping, which may discard retained inheritance elements present in the nucleotides.

## 2. Methods

The following section contains an overview of the methods used to estimate the dimensional rank of the subspaces of COG sequences and the principle angles distribution. The population sample data used in this study come primarily from the first *M_s_*=200 orthologous clusters of the NUCOCOG database reverse mapping to the NIH NCBI COG database.

### 2.1. Subspace considerations

In this work, it is preferred to obtain a reasonable estimate of the dimensions of COG subspaces at hand, since many of the common methods assume constant equal subspace dimensions in the models. The fundamental aspects considered toward estimating the rank of the subspace included review and analysis of the principal angles and singular values distributions.

#### 2.1.1. Rank estimation of a single orthologous group

Let a set of unaligned nucleotide sequences form a matrix $W_{j} \in {\mathbb C} ^{m_{j} \times M}$ for the *j*-th experiment comprising *m_j_* sequences containing, at most, *M* base pairs of a prospective orthologous cluster that is to be analyzed. To estimate the effective rank of *W_j_*, the modal selection algorithm of Yan and Pollefeys (2006) was initially used to estimate the rank *r* as follows:

\begin{align*}r_ { eff } = { { \arg \min } } _r \frac { \sigma_ {
r + 1 } ^2 }  { \sum \nolimits_ { i = 1 } ^r \sigma_ { i } ^2 } +
\kappa r \tag { 2.1 } \end{align*}

where σ_j_ is the *j*-*th* singular value and κ is a suitable constant. The foregoing method was repeated to estimate the distribution of ranks across a sample of the first *M_s_*=200 orthologous clusters from the NUCOCOG database.

#### 2.1.2. Distribution of principle angles

The principle angles between orthologous subspaces is defined *the smallest principal angle* between two subspaces *S_i_* and *S_j_*, denoted by the matrix element θ_i,j_, is defined as

\begin{align*}\cos ( \theta_ { i , j } ) { { \Delta \atop = } }
\max_ { { v } _i \in S_i , \textbf { \textit { v } } _j \in S_j }
\frac { \textbf { \textit { v } } _i ^ { \top } \textbf { \textit
{ v } } _ { j } }  { \parallel \textbf { \textit { v } } _ { i }
\parallel_ { 2 } \parallel \textbf { \textit { v } } _j \parallel_
{ 2 } } \tag { 2.2 } \end{align*}

The angular distribution was estimated by computing a subspace basis estimate for each COG and then computing the principal angles from the above equation.

#### 2.1.3. Orthologous sequences subspace basis

Let the *m_k_*-nucleotide sequences be comprised of data row vectors $s_j = [ s_{j ,  1} , s_{j ,  2} , \ldots , s_{j ,  M} ]$ whose membership is known to be the *k^th^* COG and further compose a row matrix $\{ s_1^T , s_2^T , \ldots , s_{m_{k}}^T \} _k^T = A_{k}$ such that the factorization estimate is given by

\begin{align*}\tag {2.3}
\textbf{\textit{U}}_{{{m}}_{k} \times r} {\boldsymbol\Sigma}_{r \times
r} {\textbf{\textit{V}}}_{r \times M}^{\dag} =
{\textbf{\textit{A}}}_{{{m}}_{k} \times M} [rank \ r]
\end{align*}

where $\textbf{\textit{A}}_k \in {\mathbb C}^{m_{k} \times M}$, *M* is the adjusted length of the nucleotide or protein sequence, *r* is the rank approximation of the singular value decomposition (SVD), *m_k_* is the number of sequence in the COG of interest, and † denotes the Hermitian adjoint. The subspace basis is constructed as the first-most *r* column vectors $\textbf{\textit{U}}_{m_{k} \times r} \in {\mathbb C}^{m_{k} \times r}$, and the singular value matrix $\Sigma \in {\mathbb C}^{r \times r}$ where the *k*-th subspace basis is chosen as $S_k = V^{ \dag} \in {\mathbb C}^{r \times M}$. The subspace dimension is seen to be dim(*S_k_*)=*r_k_*. In this work, the subspace basis is computed by the LSA algorithm of Yan and Pollefeys (2006).

#### 2.1.4. Sequence alignment

Prior to performing the subspace analysis, the randomly selected cluster nucleotide sequences are aligned to ensure best common basis representation. This alignment corresponds to a relative energy minimization argument and is also a best effort attempt to allow for mutations, which may include insertions and/or deletions. The multiple sequence alignment used in this study was the MAFFT code in Katoh and Standley (2013) and Chenna et al. (2003), compiled and optimized for use on the Intel i7 processor under 3.12.x baseline Linux kernel. The goal is to perform the alignment for each *W_j_* matrix as described below.

### 2.2. Probability of error estimates

In order to estimate the statistical significance of the experimental outcomes, an approximation is employed to compute the probability of the experimental data and classification outcomes. Consider the probability of randomly classifying *k*-groups into a set $\{ m_1 , \ldots , m_k \}$ where $\sum \nolimits_{i = 1}^k m_i = N_j$ items $\forall \ j \in \left[ 1 , \ldots , 30 \right]$ experiments. Let the known membership identification classification cluster vector be defined as $\textbf{\textit{c}} = \left[ c_1 , \ldots , c_N \right]$. The probability of randomly selecting the unique set of cluster memberships $c_1 , \ldots , c_{N_{j}}$ among *k*-groups from a total of *N_j_* sequences is needed for the *j*-th random experiment.

Let the random vector $S_{j} = \left\{  X_{1} , \ldots , X_{N_{j}} \right\}$ be defined as the set of random variables for the gene sequence memberships of the *j*-th experiment, and the group index element *c_i_* be defined as a mapping of the known group id such that $c_i \in [ 1 , \ldots , k ]$. The classification outcome probability is a binary event, where the probability of random success is *p_s_*=1*/k* and the probability of failure is $1 - p_s = \frac { k - 1 }  { k }$. To estimate the *p*-value tail due to one or more errors in classification results, the estimate for probability of *n_j_*-classification errors (order is not important here) and *N_j_*−*n_j_* correct for total of *N_j_* sequences of *k*-cluster groups becomes the binomial as follows:

\begin{align*}\tag{2.4}
\begin{split}
P ( s_ { j } & = \left\{ x_ { 1 } , \ldots , x_ { N_ { j } }
\right\} \mid c_ { 1 } , \ldots , c_ { N_ { j } } ;k;N_ { j } ) \\
& = \frac { N_ { j } ! }  { ( N_ { j } - n_ { j } ) !n_ { j } ! }
p_ { s } ^ { N_ { j } - n_ { j } } ( 1 - p_ { s } ) ^ { n_ { j } }
\end{split}
\end{align*}

where probability of success is *p_s_*=1*/k*. It is noted that for the experiments below, the error in the tail about the outcome is estimated as the probability of *n_j_*-errors or less. The missrate for the experiments was computed using the LSA code distribution from the online resource of Johns Hopkins University Vision, Dynamics, and Learning Lab.

### 2.3. Subspace dimensions in the model tree

In order to investigate the relationship of the subspace dimensions to the phylogenetic history, a simple randomized binary evolutionary tree was developed. Since the orthologous trees are inferred (predicted), the analysis here uses simulations of a binary randomized evolutionary tree, where each speciation branch is a random outcome of an *M*-component random vector $\textbf{\textit{v}}_i \in {\mathbb Z}_4^M$, where each component is comprised of a binary random variable product (probability of mutation) with a uniformly randomly selected nucleotide of ${\mathbb Z}_4$ (mutation).

#### 2.3.1. Simple random binary mutation tree model

In order to investigate the vector properties and singular values, a mathematical construction is used for the *k*-th nucleotide sequence vector ***s***_*k*_ in ${\mathbb Z}_4^M$ for ${\mathbb Z}_4 = \{  \overline{0} , \overline{1} , \overline{2} , \overline{3} \}$ to represent a particular sequence in the above model balanced binary random mutation tree:

\begin{align*}\tag{2.5}
\textbf{\textit{s}}_k = \mathop \sum \limits_{m =
1}^{N_{s}}\textbf{\textit{v}}_{m}C_{m , k}
\end{align*}

where ***v***_1_=***s_CA_*** is the common ancestor at the root of the tree; *v_m_*=***s***_*m*_−***s***_*parent*(*m*)_ is the *m*-th column vector of matrix *V*, which represents the mutation difference vector from the parent ***s***_*parent*(*m*)_ to child ***s***_*m*_, where the *parent*(*m*) function returns the parent branch pointer of the *m*-th child; and *C_m,k_* are scalar coefficients.

**FIG. 1.**
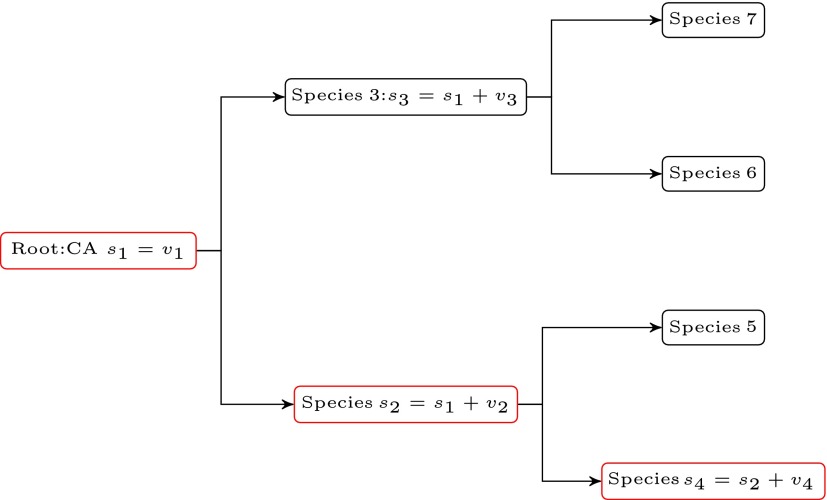
Symmetric balanced binary model tree.

An example of a simple binary model tree is shown in Figure 1. It should be noted that in reality, phylogenetic trees are generally not perfect, binary, or symmetric, due to incomplete data, missing evolutionary paths, differing branch lengths, or other reasons.

If each species branch is comprised of the sum of all speciation and mutation vectors in the path from the common ancestor, it is then possible to simulate and solve for the singular value decomposition (SVD) or eigenvalue decomposition (EVD) of the random binary tree of depth *d* having 2^*d*+1^−1=*N_s_* species vectors in ${\mathbb Z}_4^M$ ring for all *N_s_* nucleotide sequences of *M* components.

For example, compute 2^(2+1)^−1=7 speciation vectors, which also include the root common ancestor in counting. Let ***v***_1_=***s***_*ca*_ be the common ancestor, ***v***_2_=***s***_2_−***v***_1_ be the speciation mutation vector ***v***_2_ from ***s***_*ca*_ to the path leading to species ***s***_2_, and so on. By inspection of Figure 1, the resulting set of speciation nucleotide random vectors comprising the columns of matrix $S \in {\mathbb R}^{M \times N_{s}}$ may be written in terms of an upper triangular matrix form as follows:

\begin{align*}S = VC = \left[ \begin{matrix}v_{1 , 1} & v_{1 , 2}
& v_{1 , 3} & v_{1 , 4} & v_{1 , 5} & v_{1 , 6} & v_{1 , 7} \\
\vdots & v_{2 , 2} & v_{2 , 3} & v_{2 , 4} & \vdots & \vdots &
\vdots \\ & \vdots & v_{3 , 3} & v_{3 , 4} \\ & & \vdots & v_{4 ,
4} \\ & & & \vdots & \vdots \\ & & & & & \vdots \\ & & & & & &
\vdots \\ v_{M , 1} & v_{M , 2} & v_{M , 3} & v_{M , 4} & v_{M ,
5} & v_{M , 6} & v_{M , 7}\end{matrix} \right] \times \left[
\begin{matrix}1 & 1 & 1 & 1 & 1 & 1 & 1   \\ 0 & 1 & 0 & 1 & 1 & 0
& 0   \\ 0 & 0 & 1 & 0 & 0 & 1 & 1   \\ 0 & 0 & 0 & 1 & 0 & 0 & 0
\\ 0 & 0 & 0 & 0 & 1 & 0 & 0   \\ 0 & 0 & 0 & 0 & 0 & 1 & 0   \\ 0
& 0 & 0 & 0 & 0 & 0 & 1\end{matrix} \right]\end{align*}

where $\textbf{\textit{v}}_i \in {\mathbb Z}_4^M \in V \ \forall i \in [ 1 , \ldots , 7 ]$ are column vectors, and matrix $C \in {\mathbb R}^{M \times N_{s}}$ contains the selective inheritance coefficients *C_m,k_* of the column vectors *v_m_*, which comprise the species column vectors of matrix *S*. Mathematically, each random component of the mutation vector is represented by a product of a Bernoulli mutation event \begin{align*}B_{k , j}\, \sim\, f_{b} ( K;p_{b} ) = \begin{cases}p_{b} & { \rm if } \ K  = 1 , \\ \\ 1 - p_{b} & { \rm if} \ K  = 0\end{cases}\end{align*} and a uniformly distributed nucleotide random outcome *X_k,j_*∈{0,1,2,3}. Nucleotides are represented as mathematical rings as needed. Formally, let the random mutation vector be defined as

\begin{align*}\tag{2.6}
\textbf{\textit{v}}_{k} = \left[\left\{X_{k,j} B_{k,j}\right\}
\textbf{\textit{\^{e}}}_j \right]_{j = 1}^{M}.
\end{align*}

It should be noted that by the above definitions, ${\mathbb E} \left[ v_{k , j} \right] = \mu_{k , j}p_{b}$ where ${\mathbb E} \left[ X_{k , j} \right] = \mu_{k , j}$ and ${\mathbb E} \left[ B_{k , j} \right] = p_{b}$.

To simplify the analysis, it is assumed that the statistical expected magnitude of the speciation vector distance of mutation is directly proportional to the elapsed time period between the speciation node of the tree from the parent to child. In addition, a simple constant rate $\gamma_ { \phi } \sim \left( \frac { mutations }  { BP \cdot generation } \right)$ of mutation is assumed, relative to a constant molecular clock rate. Mathematically, the aforementioned assumptions allow estimation of the expected number of mutations or differences from child to parent sequence as

\begin{align*}
{\mathbb E} {\parallel} \left[\textbf{\textit{s}}_{m} -
\textbf{\textit{s}}_{parent(m)} \right]\quad { \rm mod} \ 4
{\parallel}_{p}^{1 / p} & \propto \Delta t_{m} \\
& = \;\gamma_{ \phi}g_{m} \Delta t_{m}M \\
& = \,\,\,p_{b}M\end{align*}

where $\textbf{\textit{s}}_{m} \in {\mathbb Z}_{4}^{M}$ is the *m*-th species orthologous vector, *g_m_* is the number of generations per unit time, *M* is the total number of base-pairs for the sequence, *t_m_* is the elapsed time from parent to child, and *p_b_* is the effective probability of mutation change or difference associated with the binary random speciation event over the period *t_m_*; the *p*=0 norm is employed here. Dimensional analysis of the above shows the temporal rate of mutations per speciation event to be

\begin{align*}\left[ \frac { mutations }  { BP \cdot generations
} \right] \left[ \frac { generations }  { time } \right] \left[
\frac { elapsed \ time }  { speciation \ event } \right] \left[
\frac { BP }  { sequence } \right]\end{align*}

where *BP* is the number of base-pairs in the gene sequence of interest. In the simulated results for the binary tree of this study, the mutation vectors are generated from binomial distribution where the initial probability of mutation is set to *p_b_*=0.1 for a mutation rate of 10 percent of the orthologous sequence and increased in 10 percent increments to *p_b_*=0.5.

#### 2.3.2. Stochastic phylogenetic tree model

In order to investigate the secondary hypothesis, a simplified statistical binary tree whose branches are comprised of random variables (RV) for the elapsed time was developed. Although not used here, a more extensive model may allow (1) each speciation event to be an m-fold generalization of the binary tree, which may also be an empty branch, and (2) the lengths of the speciation branches would have unequal lengths with random variable outcomes.

Using the foregoing simple mathematical model and expectation results, it is noted that the expectation of any pair of vector dot products from two randomly created binary trees *A*, *B* is statistically estimated as follows:

\begin{align*}{\mathbb E} \left[ \langle \textbf{\textit{s}}_{j}
- {\mathbb E} \left[ \textbf{\textit{s}}_{j} \right] ,
\textbf{\textit{s}}_{k} - {\mathbb E} \left[
\textbf{\textit{s}}_{k} \right] \rangle \right] \\ & \,\,\,\,
\forall \textbf{\textit{s}}_{j} \in A \hbox{;}
\,\textbf{\textit{s}}_{k} \in B ,\end{align*}

\begin{align*}{\mathbb E} \left[ \langle { \bf s}_{j} - {\mathbb
E} \left[ { \bf s}_{j} \right] , { \bf s}_{k} - {\mathbb E} \left[
{ \bf s}_{k} \right] \rangle \right] = \ 0 \,\,\, \forall \ A
\bigcap B = \emptyset \ { \rm case\ I} ,\end{align*}

\begin{align*}{\mathbb E} \left[ \langle { \bf s}_{j} - {\mathbb
E} \left[ { \bf s}_{j} \right] , { \bf s}_{k} - {\mathbb E} \left[
{ \bf s}_{k} \right] \rangle \right] \,\,\neq  \,0 \,\,\, \forall
\ A \bigcap B \neq \emptyset \ { \rm case \ II}.\end{align*}

In the above simple binary mutation tree treatment, *the IID random variable vectors components s_j_ have zero mean and are independent*. If the root common ancestor sequence vectors are randomly generated with similar IID variates, then statistically, the species vectors of tree *A* are uncorrelated with the vectors of tree *B*. Two general outcomes are noted as follows:

• case I orthologous genes of differing common ancestors lie within independent subspaces if and only if the common ancestor vectors are uncorrelated. The trees do not intersect in expectation.
•  caseII recent descendants of common ancestors may still retain nonvanishing similarity from inherited common ancestor vector components. The trees intersect.

### 2.4. Experimental approach

In order to test the subspace mutation tree relationship hypothesis, we seek to cluster sequences from the known COG database. Toward this end, two randomized experiments were conducted to test the primary hypothesis using the COG database and sets of thirty trials each containing unknown mixtures of sequences belonging to *K*∈{2, 3} orthologous groups. It should be noted that the experiments permit both false positives and false negatives. Also, the two and three groups correspond to two and three subspaces, which conceptually compare to two and three motion classification tests presented in Tron and Vidal (2007).

**FIG. 2.**
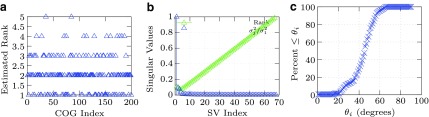
Estimates of subspace rank and distribution of principle angles. COG, Clusters of orthologous groups. **(a)** Example estimated rank (y-axis) versus COG number (x-axis) *M_s_* = 200. **(b)** Typical singular values (y-axis) distribution of a single cluster with rank estimate versus cluster eigenvector index (x-axis): *r_eff_* ≃ 4. **(c)** Typical cumulative number (y-axis) percent distribution versus principle angles (x-axis degrees) of Equation (2.7).

**FIG. 3.**
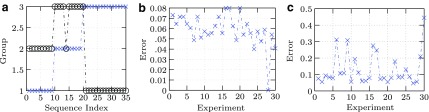
Results for M = 2 and M = 3. **(a)** Example un-randomized result for *M* = 3 COG samples clustering group (y-axis) versus experiment (x-axis); percent error = 5.7%. [*Delta-aminolevulinic acid dehydratase, Phosphoribosylamine-glycine ligase, Ribosomal protein S14*]. **(b)** Example error (y-axis) versus experiment (x-axis) using randomized samples $s_j; m_{j , 1} , m_{j , 2} , m_{j , 3}; p_{j , 1} , p_{j , 2} , p_{j , 3} \ \forall j \in [ 1 , \cdots , 30 ]$ for *M* = 2 randomly selected from *M*_*s*_ = 200 COG's; average 6 percent error. **(c)** Example error (y-axis) versus experiment (x-axis using randomized samples $s_j;m_{j , 1} , m_{j , 2}; p_{j , 1} , p_{j , 2} \ \forall j \in [ 1 , \ldots , 30 ]$ for *M* = 3 randomly selected from *M_s_* = 200 COG's; average 13 percent error.

The approach is as follows:

(1) Estimate the subspace dimensions of the first *M_s_*≃200 clusters from the NCBI COG by computing the singular value decomposition (SVD) in Akritas et al. (2006) of *j*-th cluster and computing the rank *r_j_* using Equation (2.1). Figure 2a and 2b show the estimated rank results for the first 200 clusters and a single cluster respectively. The rank of the cluster provides an estimate of the subspace dimension.
(2) Estimate inter-subspace principle angle distribution using Equation (2.2) and the following density counting functional,
  \begin{align*}f ( i , \alpha ) = \mathop \sum \limits_{j =
1}^{M_{s}} \parallel \theta_{i , j} \leq \alpha \parallel_{0} \
\forall \alpha \in [ 0 , 1 , \ldots , 90 ]. \tag{2.7}\end{align*}
  Figure 2c shows the angle distribution of the first group of *M*=200 clusters used in this study.
(3) Perform random selection for the experiment.
  (a) Draw random nucleotide sequences set $\left\{ m_{1} , m_{2} , \ldots , m_{k_{max}} \right\}$ rows from $k \in [ 2 , 3 , \ldots , k_{ \max} ]$ randomly selected clusters and compose associated sets of block row matrices $\left\{ w_1 , w_2 , \ldots , w_{k_{max}} \right\}$ where *w_k_* contains the *m_k_* rows from the *k*-th COG cluster where $\sum \nolimits_{i = 1}^{k_{max}}m_{i} = N_{j}$.
  (b) Construct the combined matrix data stack
    \begin{align*}W_{j} = \left[ \begin{matrix}w_{1} \\ w_{2} \\
\vdots \\ \\ w_{k_{max}}\end{matrix} \right] = \left[
\begin{matrix} \ldots ccgttagttcaagcc \ldots \\ \ldots
agaacagaggtag \ldots \\ \vdots \\ \\ \ldots cgccacagtacccg
\ldots\end{matrix} \right]\end{align*}
  where the rows of *W_j_* contain the nucleotide sequence set for the *j*-th experiment.
  (c) Randomize the order of rows of *W_j_* again using uniform random distribution.
(4) Align *W_j_* matrix by nucleotide sequences (rows), with preference given to global alignment algorithms using codes such as MAFFT.
  (a) Compute the subspace segmentation using LSA or other method with subspace dimensions *d*=7 and the number of known clusters (see Results section Figure 3a).
(5) Compute the misclassification rate and compare to null probability,
  (a) for each experimental sample and algorithm output set $\left\{ s_{j};c_{1} ,  \ldots , c_{N};p_{1} ,  \ldots , p_{k} \right\}$ where $\sum \nolimits_{i = 1}^{k_{max}}m_{i} = N_{j}$ are known.
  (b) Compute the *p*-values for each experiment.
(6) Go to random selection above and repeat *R*=30 times.

### 2.5. Detailed algorithm

Samples were drawn at random uniformly from the first *M_s_*=200 COG's of the nucleotide groups of the NUCOCOG reverse mapping provided by reference database Meereis and Kaufmann (2008) and Meiler et al. (2012) as used in implementation of the methods in Algorithm (1). The preliminary results reported here are in agreement with the *three-way mutual bidirectional best hits (BBH)* genomic nearest match forming the basis of a protein cluster triangle. The general method is shown in Algorithm (1), where the case for *K*=3 is similar to three-motion image segmentation. The time complexity is approximately *O*(*R*) · *O*(*lsa*()+*missclass*()+*mafft*()), where *lsa*() and *missclass*() functions are from the Johns Hopkins University LSA distribution, and *mafft*() is the MAFFT alignment program function.

## 3. Results

In this section, the average subspace dimensions are obtained from the rank estimates of the first *M*=200 clusters of our sample in this study. Representative results of the subspace clustering algorithm are presented. The singular value decomposition of simulations and rank estimates for the simple binary model tree have distributions consistent with the subspace hypothesis.

### 3.1. Estimation of parameters

The estimated rank for the first *M*=200 cluster samples is shown in Figure 2a, where the rank estimation method is further illustrated as shown in Figure 2b. Figure 2c shows the subspace angle distribution of same aforementioned cluster samples.

### 3.2. Experimental objectives

The experimental data sequence analysis methods used here are designed to evaluate the subspace algorithm hypothesis for COGs of proteins or DNA nucleotide sequences with presumed known truth from the NIH COG database. The objectives include consideration of membership, similarity, principle angles, ensemble distribution, and/or other aspects.

It is important that the experimental design allows for both false positives and false negatives, since a primary objective is to estimate the error rate and the statistical significance of the experimental results. Plots of the *p*-values for each experiment are to be computed and considered relative to the null hypothesis. The primary concern is to evaluate the stated hypothesis and related prediction: descendants of common ancestor orthologs diverge over elapsed time within subspaces. If the method demonstrates feasibility, the knowledge may be utilized within the context of future sequence analysis tools or methods. As such, the analysis herein is not a replacement for the existing COG method at this time.

### 3.3. Computational considerations

The data matrix of interest is a three-dimensional tensor of the form and size 1000–4000 (nucleotide base pairs)×50 – 100 (orthologous genus species taxon of interest)×2 – 200 (orthologous protein groups).

Several observations regarding the challenges of existing methods in COG are included below.

(1) Direct application of linear algebra is complicated by mutation events, such as insertion and deletion. However, preliminary results suggest potential for a hybrid model using subspace techniques combined with traditional methods published and demonstrated in the NIH COG series of Makarova et al. (2007).
(2) Real-world data sets are often incomplete, contain noise, and have other outliers due to errors and uncertainties and potential false positives and false negatives. These aspects generally complicate the process of modeling and inference, which may be significant. The experiments discussed in this study do not contain known nonmember outlying sequences. Nonmember outliers may be addressed in a future effort.

### 3.4. Experiments

Randomized simulations were performed using two and three clusters for *R*=30 experiments each. A typical example for a single experiment for *M*=3 is shown in Figure 3a. The average error for two orthologous gene clusters was estimated to be about 5%. The average error for three orthologous gene clusters was about 13%. Typical experimental simulation classification error results are shown in Figures 3b and c.

### 3.5. Statistical significance

Preliminary review of the estimated experimental errors and probabilities suggests that the orthologous sequences are indeed members of a common subspace, since the observed correct classification outcomes (with shown error rates) would occur due to random selection on average with probability estimate $p_{r}\precapprox 0.001$ in the case of 30 experiments using 2 orthologous clusters. The case for random successful classification (with shown error rates) of 3 orthologous clusters in 30 experiments has an average probability estimate of $p_{r} \precapprox 0.001$, but with two-fold increased success errors. The latter result suggests successful selection of three COGs at random is unlikely.

## 4. Discussion

In our small orthologous population sample study, we observe that the common ancestors are sufficiently uncorrelated as to significantly preserve the inherited vector components that allow the LSA (Zappella et al., 2011) algorithm to perform the subspace classification of descendants relative to their common ancestors. A larger study would be needed to ascertain the limiting degree of applicability of the observed distinctiveness of the common ancestor orthologous sequences.

### 4.1. Supplemental results for simple model tree

This section includes results from simulation and analysis of the simple binary model tree. The characteristics of rank, tree depth, and number of nodes are consistent with the main hypothesis and are included here for reference. It should be noted that operations such as dot products and projections are generally performed after alignment. Also, the *ℓ*^2^ or *ℓ*^0^ distances have been used in the literature (Kim and Lee, 2006; Viswanath and Madabhushi, 2012).

#### 4.1.1. Idealized model tree

**FIG. 4.**
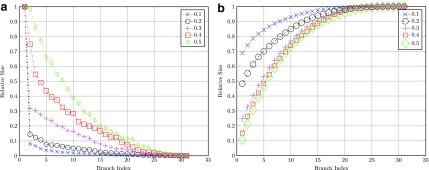
Simple binary mutation tree simulations. **(a)** Symmetric balanced binary model tree $N_s = 31 , \sigma^2_k / \sigma^2_1$ versus mutation rate. **(b)** Cumulative sum normed of symmetric balanced binary model tree $N_s = 31 , \sum \nolimits^k_{j = 1} \sigma^2_j / \sum \nolimits^{N_{s}}_{k = 1} \sigma^2_k$ versus mutation rate.

The simple randomized binary mutation tree was generated for mutation rates *p_b_* ranging from 0.1 (10%) to 0.5 (50%), and the singular values were computed. The simple model simulation results are shown in Figure 4a. The distribution of singular values is sharply concentrated for 0.1 mutation rate but spreads out significantly for the higher mutation rate. These results are consistent with increasing effective rank versus mutation rate. The cumulative relative sums over the singular values of the same results are shown in Figure 4b. The effective rank may be estimated by the 90% area under the curve, which gives ranks ranging from circa 8 to 18, with the larger ranks correlating with larger mutation rates.

#### 4.1.2. Singular values versus binary tree depth

**FIG. 5.**
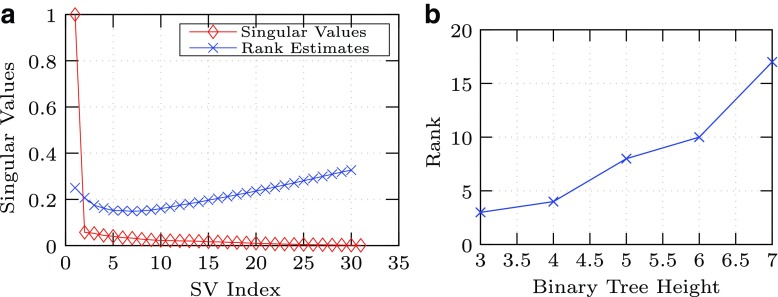
Estimation of group rank. **(a)** Example determination of rank for symmetric balanced binary phylogenetic model tree with random mutation vectors (estimated *rank* = 6, *h* = 4, 31 × 64 sequences by nucleotides, and 10 percent average branch mutation of nucleotides. **(b)** Estimated SVD rank of a simulated symmetric balanced binary phylogenetic model tree with random mutation vectors. Rank (y-axis) versus binary tree height (x-axis) (13 × 64 sequences by nucleotides and 10 percent average branch mutation of nueclotides).

Simulations using the random binary tree evolution model were run over a range of depths. For each complete balanced random tree, the rank was estimated using Equation (2.1), as shown in Figures 2a and 5a. The dependence of estimated rank versus binary tree depth is noted in Figure 5b.

#### 4.1.3. Singular values versus mutation rates

The simulated results for the simple binary mutation tree are shown in Figures 4a and b. The rank of the tree for the given mutation may be estimated at the 90% level in the cumulative sum of the singular values normalized for the two cases here, *N_s_*=[31, 15]. It is seen that the rank of the tree is a function of the tree depth *d* and mutation rate γ_*φ*_ as follows:

\begin{align*}r_{eff}\, \sim \, f ( d , \gamma_{ \phi} ). \tag{4.1}\end{align*}

Since the mutation time lengths are constant in this simple model, it is expected that a better model would allow for random mutation time.

#### 4.1.4. Subspace relationships in the simple model tree

The dimensions of the subspaces are observed to be dependent upon similarities among the sequences. In particular, it is observed that the mutation rate product with the number of sequence replication is proportional to the estimated rank dimensions of the binary tree. This is consistent, since a tree with very high rate of mutations would produce species that were all significantly different, and the full rank of the sequence matrix would be required for the subspace representation. In addition, a perfect binary tree shows increasing rank versus depth, as illustrated in the simulations presented.

The subspace nature of orthologous groups in general is believed to have a connection within the molecular aspects of divergent evolutionary processes. In particular, the simple mathematical model binary tree simulations indicate descendants from two uncorrelated unique common ancestors are statistically unlikely to intersect, and therefore, the subspaces of binary trees are not expected to intersect. It is important to note that statistically, the mathematical model allows exceptions and variance about the mean, which is consistent with exceptions that are expected in nature and biology. However, the reader should be mindful that the binary tree is generally too simple for most orthologous sequence relationships, and is primarily used here as a means for analysis.

#### 4.1.5. Model tree simulations

Based upon the results in Figure 1, the estimated dimensional rank of the subspace *S_j_* follows a relation with the height of the tree, *h*, as anticipated, which is observed to be a monotonically increasing function over the data range. The subspace dimension for each orthologous group could be the estimated value as determined empirically using Equation 4.1.

In addition, the simulation data are consistent with the following observation: rank estimates for the perfect complete binary tree are dependent upon the rate of mutation and the elapsed time, which give rise to the length of the branches. The larger the rate of mutations per generation for a given unit of time, the more quickly descendants diverge from the common ancestor, and the more significant the members of the branches become in the singular value decomposition, thereby increasing the rank approximation value [Eq. (2.1)]. In summary, the observed results support the hypothesis relating the mutation events in the phylogenic tree to the subspace constraint.

## 5. Conclusions

The experimental results are consistent with the hypothesis that similar genes of various related organisms lie within relatively small subspaces. Randomized experimental simulations were performed using two and three clusters for *R*=30 experiments each. The average *p*-values of each series were found to be *p*≪0.001 and support the subspace hypothesis. As a consequence, the results suggest that multiple orthologous genes comprise a union of subspace representation. It is expected that other genes of an organism's genome do not lie closer to the orthologous cluster subspace than does the COG member. However, the degree of uniqueness should be verified independently. Future efforts should improve methods of alignment and selection of focal regions. For example, it is expected that use of critical regions, such as maximum entropy or centroids of the sequences, would provide faster performance with regard to alignment and subspace segmentation. Also, early testing using GPCA showed promise for improved performance, but further investigation is needed. It is envisioned that the use of the subspace clustering algorithms and concepts may provide an additional means (in addition to NIH COG and OrthoMCL) for theoretical and applied classification of related sequences.

## 6. Appendix

A brief overview and discussion of subspace clustering and related theory is presented below with references from the literature.

### 6.1. The subspace theoretic problem

**Problem 1.** The general subspace clustering or segmentation problem can be stated as follows: Let ${ \cal U} = \bigcup \nolimits_{i = 1}^{M}S_{i}$ where $\left\{ S_{i} \subset{ \cal B} \right\} _{i = 1}^{M}$ is a set of subspaces of a Hilbert space or Banach space ${ \cal B}$. Let ${ \bf W} = \left\{ \textbf{\textit{w}}_{j} \in { \cal B} \right\}  _{j = 1}^{N}$ be a set of data points drawn from ${ \cal U}$. Then,

(1) determine the number of subspaces *M*,
(2) determine the set of dimensions $\left\{ d_{i} \right\}  _{i = 1}^{M}$,
(3) find an orthonormal basis for each subspace *S_i_*,
(4) collect the data points belonging to the same subspace into the same cluster.

### 6.2. Subspace segmentation methods

Subspace clustering may be viewed as multiple independent nonintersecting (except at zero) low-rank spaces amenable to singular value decomposition or principle components in linear algebra. The technique has shown success in areas such as face recognition and motion detection.

Methods in the literature (Vidal, 2010) generally fall into four categories:

• algebraic methods—generalized principle component analysis (GPCA) and reduced row echelon form (RREF);
• sparsity methods—sparse subspace clustering (SSC) and low rank representation (LRR);
• local neighborhood methods—local subspace affinity (LSA), nearness to local subspace (NLS), and spectral curvature clustering (SCC);
• iterative and statistical methods—random sample consensus (RANSAC) and agglomerative lossy compression (ALC).

Most of the methods incur significant numerical computational complexity, owing to the number of unknown quantities of the set characteristics, such as dimensions, bases, and members, for general problem domains. The sparsity methods often require the most computational cost relative to the other methods.

References for solving Problem 1 in the literature include sparsity methods (Eldar and Mishali, 2009; Elhamifar and Vidal, 2009; Elhamifar and Vidal, 2010); algebraic methods (Vidal et al. 2005; Tron and Vidal, 2007); iterative and statistical methods (Kanatani and Sugaya, 2003; Aldroubi et al., 2009; Tseng, 2000; Fischler and Bolles, 1981; Silva and Costeira, 2008; Aldroubi and Zaringhalam, 2009); and spectral clustering methods (Lauer and Schnorr, 2009; Chen and Lerman, 2009). These methods and applications on subspace clustering are reviewed and discussed in Vidal (2010).

**Table:** 

**Algorithm 1:** Subspace Clustering Experimental Algorithm for Randomly Selected Data from *K*=3 Orthologous Groups
**Require:** Input *M_s_*=200 known clusters of orthologous groups (COG) gene data clusters of nucleotides from which to form the experiment matrices $W_{j}^{T} = \{ w_{j , 1} , \ldots , w_{j , k_{max}} \} ^{T} \in \mathbb{R} ^{Q \times N_{j}}$ comprised of noiseless data measurements containing an ensemble randomized mixture of *k_max_*-known subspaces each of dimensions *d* for data originally of block rows $\textbf{\textit{w}}_{j , k} \in \mathbb{R}^{n_{j} \times Q}$ of sequences, having *n_j_* sequence rows and *Q* columns of basepairs (padded if needed).
1: set *R*=30 (e.g., set *k_max_*=*K*=3 for three COGs)
2: **for** for $j = 1 , 2 , \ldots , R$**do**
3: **for** for $k = 1 , 2 , \ldots , K$**do**
4: sample *m_j,k_*-data sequences at random uniformly from the *k*-th random uniformly selected COG and create row block matrices $w_{j , k} \in \mathbb{R}^{m_{j , k} \times s_{j , k}}$ where *s*_*j,k*_ is the greatest block length of the nucleotide sequences
5: set $$W_{j} = \left[ \begin{matrix} w_{j , 1} \\ w_{j , 2} \\ \vdots \\ w_{j , K}\end{matrix} \right]$$ and perform blind randomization of the rows to obtain $\tilde{W_{j}}$ and but first save the truth mapping of row vectors as column vector $r_{j} \in \mathbb{R}^{N_{j}}$ where $N_{j} = m_{j , 1} + m_{j , 2} , \ldots , + m_{j , K}$.
6: align ensemble *W_j_* using MAFFT program
7: use LSA subspace segmentation algorithm to classify the randomized $\tilde{W_{j}}$ matrix into a group membership column vector $M_{j} \in \mathbb{R}^{N_{j}}$.
8: compute the miss-rate *n_j_* vector by comparing errors between truth *r_j_* and classification result *M_j_*
9: compute the probability of obtaining *n_j_* errors or less in *N_j_* outcomes due to random occurrence for statistical significance estimates of the p-value.
10: **end for**
11: **end for**
